# Study on the Mechanism of Energy Dissipation in Hemispherical Resonator Gyroscope

**DOI:** 10.3390/s25010074

**Published:** 2024-12-26

**Authors:** Lishan Yuan, Ning Wang, Ronghao Xie, Zhennan Wei, Qingshuang Zeng, Changhong Wang

**Affiliations:** Space Control and Inertial Technology Research Center, Harbin Institute of Technology, Harbin 150080, China; lyuan@stu.hit.edu.cn (L.Y.); ning.w@stu.hit.edu.cn (N.W.); 22s104141@stu.hit.edu.cn (R.X.); wzn@hit.edu.cn (Z.W.); cwang@hit.edu.cn (C.W.)

**Keywords:** hemispherical resonator gyroscope, energy transfer path, dissipation mechanism, quality factor

## Abstract

The hemispherical resonator gyroscope is a gyroscope based on the principle of Coriolis vibration, widely used in inertial measurement systems of spacecraft. This article decomposes the gyroscope into two parts: the resonator shell and the gyroscope head, establishes the energy dissipation mechanism of the gyroscope, and conducts experimental verification. Firstly, based on the working principle of the gyroscope, a mechanical analysis model of the hemispherical resonator gyroscope head with a resonator spherical shell containing quality defects under second-order vibration state was established. The unbalanced force applied by the resonator spherical shell to the hemispherical resonator gyroscope head was analyzed, and the energy transfer path and dissipation mechanism from the spherical shell to the hemispherical resonator gyroscope head were explained. Finally, through the constructed testing platform, the circumferential quality factor test of the hemispherical resonator gyroscope before and after assembly was completed according to the designed experimental plan, and the consistency between theory and experimental phenomena was verified experimentally.

## 1. Introduction

The hemispherical resonator gyroscope (HRG) is a new type of vibratory gyroscope that first appeared in the 1960s and is known for its high accuracy, long life, wear-free performance and extremely high reliability [1]. Compared with traditional mechanical and optical gyroscopes, the HRG has a simpler structure with no rotors or moving parts. Its resonator is made of low-damping and temperature-stable quartz, resulting in lower cost. The characteristics of this HRG make it the best candidate for stability, precise pointing, spacecraft navigation, strategic precision systems, oil well exploration and planetary exploration [2,3,4]. At present, research on HRGs has shown their potential in space applications and technological performance, and these research results can further promote the development of related fields [5,6].

At present, the working mode of hemispherical resonator gyroscope can be divided into force feedback mode and full angle mode. The working point of HRG with force feedback is constant, so only the drift error of the working point needs to be calibrated and compensated. Each position in the circumferential direction of the HRG resonator in full angle mode is a working point, so it is necessary to correct the drift error at each position in the circumferential direction of the resonant cavity. According to the fixing method of hemispherical resonator gyroscope, it can be divided into two: end fixing scheme and single end fixing scheme. Using a double-ended fixing scheme, the unbalanced mass of the hemispherical shell makes it difficult to drive the vibration of the support rod, resulting in constant standing wave drift error and minimal energy dissipation. The single-ended fixed scheme, with the unbalanced mass of the spherical shell, can easily drive the vibration of the support rod, resulting in uneven quality factor and unstable standing wave drift rate, which greatly affects energy dissipation. This article studies a hemispherical resonator gyroscope with a single-ended fixed full angle mode. In summary, it can be concluded that changes in the working mode and fixing method result in energy dissipation in the hemispherical resonator gyroscope. In order to achieve the characteristics of miniaturization and smaller volume, the three-piece gyroscope has been transformed into a two-piece gyroscope, removing the top rod of the resonator and simplifying the form of the electrode, resulting in new problems. The force feedback hemispherical resonator gyroscope is basically in the form of a three-piece set, with separate excitation and detection, consisting of 16 electrodes and fixed at both ends. This article adopts a time-division multiplexing approach to reduce volume, using only eight electrodes for excitation and detection. This structure amplifies the influence of the first three harmonics of quality defects on standing wave drift, which are important errors introduced during the processing of hemispherical resonators.

The hemispherical resonator gyroscope consists mainly of a hemispherical resonator and a flat electrode. The flat electrode provides a fixed anchor point for the hemispherical resonator and is also made of fused silica. Its upper surface also forms an electrode by preparing a chromium-gold composite metal film [7,8,9]. Using the capacitance formed by the electrode and the end face of the resonator, standing wave excitation and detection of the resonator can be achieved [10]. The flat electrode hemispherical resonator gyroscope uses a mushroom-shaped resonator with only a single support rod, while the traditional structure hemispherical resonator gyroscope uses a PSI-shaped resonator with double support rods [11]. The damping inhomogeneity of the hemispherical resonator gyroscope is mainly caused by the uneven mass distribution inside the gyroscope, and the damping inhomogeneity can also lead to the angular drift of the gyroscope [12]. In practical engineering, the mass distribution of the hemispherical resonator may be inhomogeneous due to morphological errors and internal defects during processing, which can seriously affect the natural frequency and operating modes of the gyroscope. In order to improve the accuracy and performance of the gyroscope, Song simulates and analyses the ideal state and the state of inhomogeneous mass distribution by setting up the partial differential equations of the hemispherical resonator and constructing a three-dimensional model using ANSYS15.0 (64 bit, John, Swanson, PA, USA) software to simulate and analyze the ideal state and the inhomogeneous mass distribution modes, which in turn suppresses frequency splitting [13]. Considering that the unevenness of the damping can lead to the angular drift of the gyroscope, Guo proposed a recursive least squares method to identify the unevenness of the damping and calculate the optimal parameters through simulation to eliminate the angular drift caused by the unevenness of the damping [14]. In addition, concentric errors during assembly can also lead to excitation coupling errors in the force-balance mode, which can lead to mutual coupling between the drive and sensing loops, reducing the stability of the drive and introducing additional zero offset drift. Ruan can effectively compensate for such errors by identifying the coupling coefficients of the excitation forces and calibrating them to significantly improve the stability of the gyroscope [15]. The quality factor of the hemispherical resonator gyroscope is mainly affected by material damping, surface loss, thermoelastic damping and anchoring loss [16,17,18,19]. An effective simulation method for obtaining the temperature-dependent thermoelastic damping quality factor of mechanical modes in complex MEMS gyroscopes has been reported in the literature [20], which showed that the temperature dependence of the TEDs has a significant impact on the overall quality factor of the higher order modes in the application-relevant temperature range. In particular, the anchor point loss is strongly dependent on the properties of the shell, stem and substrate, and any imperfection in the shell or misalignment between the shell and stem will significantly increase the anchor point loss [18]. In the literature [21], the effect of assembly errors and manufacturing defects on anchor losses in high quality factor resonators has been analyzed. It was found that even small geometric deviations and structural defects can lead to large anchor losses, and that the anchor losses of hemispherical resonators are highly dependent on the characteristics of the shell, rod and backing [22]. A study in the literature [23] describes that the unbalanced mass of the shell leads to a forced vibration of the support beams in the operating mode of the resonator, and the mechanical energy of the shell is dissipated through the support beams, resulting in an asymmetric error in the Q-factor of the resonator in the circumferential direction. References [24,25,26] describe the first and third harmonic components of the unbalanced mass that cause the center of mass of the resonator to shift in the operating mode, which in turn affects the performance of the gyroscope. To improve the angle bias error caused by damping asymmetry, Fan et al. designed a gain self-correction scheme based on the variation of the scale factor due to the quality factor asymmetry [27]. The coupling effect between the shell and the support rod not only causes anchoring loss and damping asymmetry error, but also leads to the vibration axis drift in the resonator’s eigenmode, which seriously affects the gyroscope’s operational performance.

In summary, there are still many deficiencies in the analysis of the mechanism of the effect of uneven damping of the hemispherical resonator gyroscope head on the unevenness of its circumferential quality factor, which are mainly reflected in the following three aspects: (1) The mechanical analysis model of the quality defect of the resonator spherical shell to the hemispherical resonator gyroscope head has not yet been established. (2) The transfer mechanism of energy dissipation from the resonator shell to the hemispherical resonator gyroscope head is not yet clear. (3) The mathematical relationship between the quality defects of the resonator spherical shell and its quality factor inhomogeneity has not been established. This article first divides the gyroscope into two parts based on its basic structure and working principle to study its energy dissipation mechanism. Then, a mechanical model of the gyroscope head and resonator shell with quality defects was established, and the unbalanced force exerted by the resonator shell on the gyroscope head was analyzed. On this basis, the energy transfer path and dissipation mechanism were explained and analyzed. Finally, testing platforms for the non-uniformity of circumferential quality factors of hemispherical resonators and hemispherical resonator gyroscopes were built separately. The experiment verified the rationality of the theory. This provides a reference for undeveloped hemispherical resonator gyroscopes with high Q values and low damping non-uniformity.

## 2. Methods

### 2.1. Basic Structure and Working Principle of Hemispherical Resonator Gyroscope

**Figure 1:**
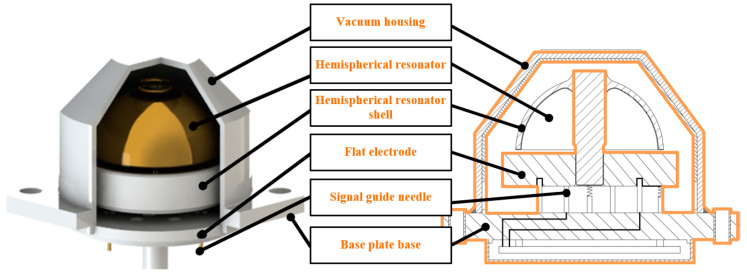
Schematic diagram of hemispherical resonator gyroscope.

As shown in Figure 1, the main components of the structure of the hemispherical resonator gyroscope include a gyroscope case, a hemispherical resonator, a flat plate electrode, a base plate base, and a signal lead pin. Among them, the hemispherical resonator and the flat plate electrode constitute the core components of the gyroscope. After precision machining and chemical etching of the hemispherical resonator, a continuous, electrically conductive chromium-gold composite metal film is uniformly formed on the inner surface, the end face along the lip and the surface of the support rod by magnetron sputtering. This process ensures that the thickness of the film remains consistent from site to site. For flat electrode substrates, the upper surface is treated with a similar process to form a conductive metal film. Subsequently, the metal film on the electrode surface is etched using laser technology to create eight identically shaped and independent electrodes. When these electrodes are fixedly connected to the hemispherical resonator by an assembly welding process, the overlapping area between the lip along the end face of the resonator and the electrodes in the vertical direction will form a parallel-plate capacitor, which is one of the key structures for realizing high-precision measurements.

### 2.2. Equivalent Model of a Hemispherical Resonator Gyroscope

**Figure 2:**
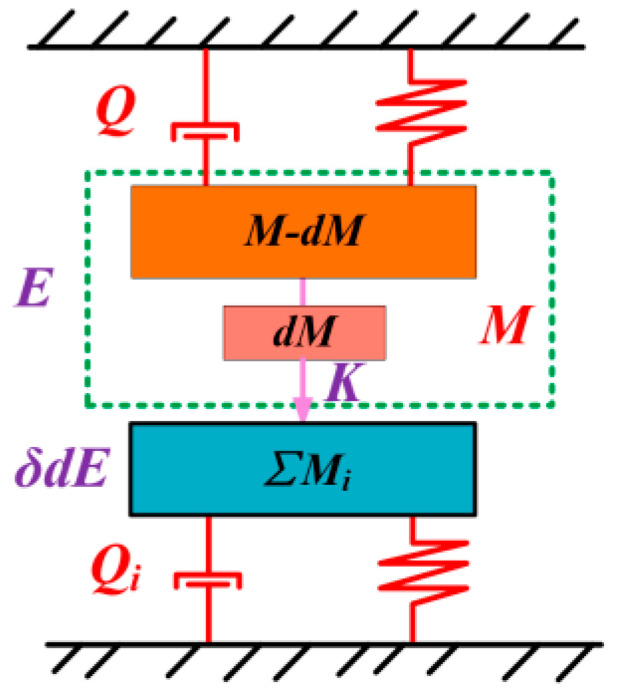
Equivalent model of hemispherical resonator gyroscope energy dissipation.

As shown in Figure 2, it is represented as an equivalent model of the energy dissipation of the hemispherical resonator gyroscope, *Q* represents the energy dissipation of the resonator itself, while *Q_i_* represents the energy dissipation of the mechanical connection. In this case, the modal mass of the hemispherical shell portion of the hemispherical resonator gyroscope can be denoted as *M*. *dM* is the asymmetric non-uniform modal mass on the shell. The non-uniform mass *dM* transfers energy to the neighboring modes of the four-wave amplitude vibration mode. Assume that the energy on *dM* is *dE*. The energy *dE* of the non-uniform mass *dM* is proportional to the total energy *E* of the hemispherical resonator:

(1) $$dE=E\times \frac{dM}{M}$$

The energy *dE* possessed on the non-uniform mass *dM* is partially transferred to the other modes of the hemispherical resonator, and then the other order mode of the hemispherical resonator acquires the energy as *δ_i_dE*:

(2) $$\delta_{i}dE=K\cdot dE$$

where *K* is the ratio coefficient of the energy transfer between modes affecting the hemispherical resonator.

The non-uniform mass *dM* and the ratio coefficient *K* are the key factors affecting the vibration quality factor of hemispherical resonators. In order to prevent energy dissipation, in addition to reducing the unbalanced mass *dM*, it is necessary to reduce the magnitude of the *K* value in the design to improve the quality factor. Reducing the value of *K* is mainly to avoid the intrinsic frequency of other modes close to the resonator frequency of the working mode in the hemispherical shell and the anchor connection design modal vibration shape nodes, as far as possible to avoid the hemispherical shell energy dissipation through the anchor.

As shown in Figure 2, the hemispherical resonator gyroscope head can be treated as a multiple single-degree-of-freedom vibration system, which can be expressed as:

(3) $$\left\{\begin{array}{c} {\ddot{x}}_{1}+2\zeta \omega_{1}\dot{x}+\omega_{1}^{2}x=A_{f}\sin\omega t\\ {\ddot{x}}_{2}+2\zeta \omega_{2}\dot{x}+\omega_{2}^{2}x=A_{f}\sin\omega t\\ \cdots \\ {\ddot{x}}_{n}+2\zeta \omega_{n}\dot{x}+\omega_{n}^{2}x=A_{f}\sin\omega t \end{array}\right.$$

where *ζ* denotes the damping ratio, *ω_n_* denotes the frequency of the hemispherical resonator gyroscope watch head, *A_f_* denotes the amplitude of the excitation force, and *ω* denotes the frequency of the resonator.

Therefore, viewing the overall housing as a multiple single-degree-of-freedom vibration system, externally applied stresses will cause the frequency and damping ratio of the housing to change, thus causing the frequency of the head housing to approach the resonator frequency of the operating mode of the hemispherical resonator sub-sphere housing, leading to energy dissipation in the hemispherical resonator gyroscope.

### 2.3. Establishment of Hemispherical Resonator Gyroscope Coordinate System

The coupling effect between the resonator shell and the support rod not only causes anchoring loss and damping asymmetry error, but also leads to vibration axis drift of the resonator characteristic mode, seriously affecting the working performance of the gyroscope. Figure 1 shows the structural distribution of a hemispherical resonator gyroscope. Divide the entire gyroscope into two parts: one is a hemispherical resonator shell; the other one is represented by an orange line in the figure, which includes the support rod of the hemispherical resonator, the flat electrode, the base plate base, the signal guide needle, and the vacuum housing, and is named as a whole hemispherical resonator gyroscope head.

**Figure 3:**
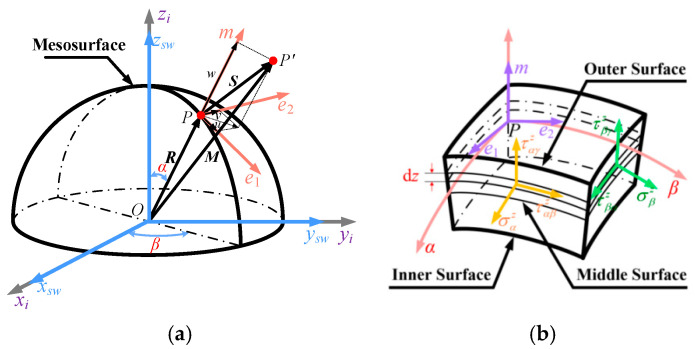
Deformation and stress of the resonator. (**a**) Deformation of the mesosurface. (**b**) Stress in the micrometric body.

In order to model the dynamics of the hemispherical resonator, the resonator is simplified as a hemispherical shell with uniform wall thickness, and the corresponding coordinate system is established to analyze the transfer relationship of the positional angular motion parameters, as shown in Figure 3.

➀ Spatial reference coordinate system $O–x_{i}y_{i}z_{i}$: the coordinate origin *O* is located in the center of the bottom surface of resonator shell, the $z_{i}$-axis is perpendicular to the bottom surface of the shell and coincides with the rotational axis of the resonator, and the $x_{i}$-axis and the $y_{i}$-axis are located in the bottom surface of the shell and are perpendicular to each other.
➁ The overall coordinate system of the hemispherical resonator spherical shell $O–x_{sw}y_{sw}z_{sw}$: the coordinate origin *O* is located at the center of the bottom surface of the resonator spherical shell.
➂ Coordinate system of orthogonal curves of the hemispherical resonator P–αβz: the coordinate origin is *P* inside the surface of the spherical shell of the resonator, the *z*-axis is perpendicular to the surface of the spherical shell, and the *α*-axis and *β*-axis are located inside the surface of the spherical shell and are orthogonal to each other.
➃ The hemispherical resonator shell local coordinate system $P–e_{1}e_{2}m$: the coordinate origin *P* is located in the middle surface of the resonator shell, and the $e_{1}$-axis, $e_{2}$-axis and *m*-axis coincide with the tangent direction of the *α*-axis, *β*-axis and *z*-axis, respectively.

In order to both characterize the circumferential mass distribution of the spherical shell of the resonator and to enhance the ease of mathematical modeling of the resonator as well, the density parameter *ρ* is chosen here as the independent variable and expanded into a Fourier series about the circumferential angle *β* of the spherical shell, denoted as:

(4) $$\rho \left(\beta \right)=\rho_{0}\left\{1+\sum_{i=1}^{\infty }\varepsilon_{i}\cos\left[i\left(\beta -\beta_{i}\right)\right]\right\}$$

where $\rho_{0}$ represents the mean value of the circumferential density of the resonator spherical shell; $\varepsilon_{i}$ and $\beta_{i}$ represent the relative amplitude and phase of the *i*th harmonic component of the inhomogeneous circumferential density distribution of the resonator spherical shell, respectively.

### 2.4. Mechanical Analysis Model of Hemispherical Resonator Gyroscope

From Figure 3, the transformation matrix from the local coordinate system $P–e_{1}e_{2}m$ of the resonator spherical shell to the overall coordinate system $O–x_{sw}y_{sw}z_{sw}$ of the resonator spherical shell is:

(5) $$C_{e}^{h}=\left[\begin{array}{ccc} \cos\alpha \cos\beta & -\sin\beta & \sin\alpha \cos\beta \\ \cos\alpha \sin\beta & \cos\beta & \sin\alpha \sin\beta \\ -\sin\alpha & 0 & \cos\alpha \end{array}\right]$$

When the resonator spherical shell is in the second-order vibration mode, the linear acceleration of any point on the curved surface in the spherical shell in the curvilinear coordinate system *α*, *β* and *z* direction is:

(6) $$\left[\begin{array}{c} a_{u}\\ a_{v}\\ a_{w} \end{array}\right]=-\frac{A_{l}\omega_{0}^{2}}{2}\left[\begin{array}{c} U\left(\alpha \right)\cos\left(2\beta -2\vartheta \right)\\ -V\left(\alpha \right)\sin\left(2\beta -2\vartheta \right)\\ W\left(\alpha \right)\cos\left(2\beta -2\vartheta \right) \end{array}\right]\sin\omega_{0}t$$

where $A_{l}$ is the vibration amplitude along the lip of the spherical shell of the resonator; *ω*_0_ is the intrinsic angular frequency of the resonator, ϑ is the azimuth angle of the standing wave, and *t* is the vibration time of the resonator.

Through coordinate transformation, the linear acceleration ***a_F_*** of any point on the curved surface of the resonator spherical shell along the directions of the resonator coordinate system $x_{sw}$, $y_{sw}$ and $z_{sw}$ can be obtained as:

(7) $$\begin{array}{l} a_{F}={\left[\begin{array}{c} a_{F_{x_{sw}}}\\ a_{F_{y_{sw}}}\\ a_{F_{z_{sw}}} \end{array}\right]}^{T}=C_{e}^{h}{\left[\begin{array}{c} a_{u}\\ a_{v}\\ a_{w} \end{array}\right]}^{T}=-\frac{A_{l}\omega_{0}^{2}}{2}\left[\begin{array}{l} U\left(\alpha \right)\cos\alpha \cos\beta \cos\left(2\beta -2\vartheta \right)+\\ U\left(\alpha \right)\cos\alpha \sin\beta \cos\left(2\beta -2\vartheta \right)-\\ -U\left(\alpha \right)\sin\alpha \cos\left(2\beta -2\vartheta \right)+ \end{array}\right.\\ \left.\begin{array}{l} V\left(\alpha \right)\sin\beta \sin\left(2\beta -2\vartheta \right)+W\left(\alpha \right)\sin\alpha \cos\beta \cos\left(2\beta -2\vartheta \right)\\ V\left(\alpha \right)\cos\beta \sin\left(2\beta -2\vartheta \right)+W\left(\alpha \right)\sin\alpha \sin\beta \cos\left(2\beta -2\vartheta \right)\\ W\left(\alpha \right)\cos\alpha \cos\left(2\beta -2\vartheta \right) \end{array}\right]\sin\omega_{0}t \end{array}$$

When the hemispherical shell with inhomogeneous mass is in the second-order vibrational mode, according to Equation (7) as well as Equation (4), the hemispherical resonator gyroscope head is subjected to a force along the $z_{sw}$ direction as:

(8) $$\begin{array}{cc} F_{z_{sw}}\; & =\int_{R_{m}-\frac{h}{2}}^{R_{m}+\frac{h}{2}}\int_{0}^{2\pi }\int_{0}^{\frac{\pi }{2}}\rho \left(\beta \right)\cdot a_{F_{z_{sw}}}\cdot r^{2}\sin\alpha d\alpha d\beta dr\\ & \approx -\frac{A_{l}\omega_{0}^{2}\pi \varepsilon_{2}\rho_{0}hR_{m}^{2}}{2}\int_{0}^{\frac{\pi }{2}}\left[U\left(\alpha \right)\sin\alpha -W\left(\alpha \right)\cos\alpha \right]\sin\alpha d\alpha \cdot \cos\left(2\vartheta -\right.\left.2\beta_{2}\right)\sin\omega_{0}t \end{array}$$

where *h* is denoted as the thickness of the hemispherical shell, *R_m_* is denoted as the mid-surface radius of the resonator spherical shell.

Equation (8) shows that when the resonator spherical shell is in the second-order vibration mode, the second harmonic of its mass defect causes the hemispherical resonator gyroscope head to initiate forced vibration in the $z_{sw}$ direction.

When the hemispherical shell with inhomogeneous mass is in the second-order vibration mode, according to Equation (7) as well as Equation (4), the hemispherical resonator gyroscope head is subjected to a force along the $x_{sw}$ direction as:

(9) $$\begin{array}{cc} F_{x_{sw}}\; & =\int_{R_{m}-\frac{h}{2}}^{R_{m}+\frac{h}{2}}\int_{0}^{2\pi }\int_{0}^{\frac{\pi }{2}}\rho \left(\beta \right)\cdot a_{F_{x_{sw}}}\cdot r^{2}\sin\alpha d\alpha d\beta dr\\ & =-\frac{A_{l}\omega_{0}^{2}\pi \rho_{0}hR_{m}^{2}}{4}\left\{\int_{0}^{\frac{\pi }{2}}\left[U\left(\alpha \right)\cos\alpha +V\left(\alpha \right)+W\left(\alpha \right)\sin\alpha \right]\sin\alpha d\alpha \cdot \right.\varepsilon_{1}\cos\left(2\vartheta -\beta_{1}\right)\\ & +\left.\int_{0}^{\frac{\pi }{2}}\left[U\left(\alpha \right)\cos\alpha -V\left(\alpha \right)+W\left(\alpha \right)\sin\alpha \right]\sin\alpha d\alpha \cdot \varepsilon_{3}\cos\left(2\vartheta -3\beta_{3}\right)\right\}\sin\omega_{0}t \end{array}$$

Which is available according to the literature [6]:

(10) $$\frac{\int_{0}^{\frac{\pi }{2}}\left[U\left(\alpha \right)\cos\alpha +V\left(\alpha \right)+W\left(\alpha \right)\sin\alpha \right]\sin\alpha d\alpha }{\int_{0}^{\frac{\pi }{2}}\left[U\left(\alpha \right)\cos\alpha -V\left(\alpha \right)+W\left(\alpha \right)\sin\alpha \right]\sin\alpha d\alpha }=3$$

Collating Equation (9) gives:

(11) $$\begin{array}{ll} F_{x_{sw}}\; & =-\frac{A_{l}\omega_{0}^{2}\pi \rho_{0}hR_{m}^{2}}{4}\int_{0}^{\frac{\pi }{2}}\left[\begin{array}{l} U\left(\alpha \right)\cos\alpha \\ -V\left(\alpha \right)+W\left(\alpha \right)\sin\alpha \end{array}\right]\sin\alpha d\alpha \cdot \\ & \left[3\varepsilon_{1}\cos\left(2\vartheta -\beta_{1}\right)+\right.\left.\varepsilon_{3}\cos\left(2\vartheta -3\beta_{3}\right)\right]\sin\omega_{0}t \end{array}$$

Equation (11) shows that when the resonator spherical shell is in the second-order vibration mode, the first harmonic and the third harmonic of its mass defect will cause the hemispherical resonator gyroscope head to initiate forced vibration in the $x_{sw}$ direction.

Similarly, when the hemispherical shell with inhomogeneous mass is in the second-order vibration mode, according to Equation (7) as well as Equation (4), the hemispherical resonator gyroscope head is subjected to a force along the $y_{sw}$ direction as:

(12) $$\begin{array}{cc} F_{y_{sw}}\; & =\int_{R_{m}-\frac{h}{2}}^{R_{m}+\frac{h}{2}}\int_{0}^{2\pi }\int_{0}^{\frac{\pi }{2}}\rho \left(\beta \right)\cdot a_{F_{y_{sw}}}\cdot r^{2}\sin\alpha d\alpha d\beta dr\\ & =\frac{A_{l}\omega_{0}^{2}\pi \rho_{0}hR_{m}^{2}}{4}\int_{0}^{\frac{\pi }{2}}\left[U\left(\alpha \right)\cos\alpha -V\left(\alpha \right)+W\left(\alpha \right)\sin\alpha \right]\sin\alpha d\alpha \cdot \\ & \left[3\varepsilon_{1}\sin\left(2\vartheta -\beta_{1}\right)-\right.\left.\varepsilon_{3}\sin\left(2\vartheta -3\beta_{3}\right)\right]\sin\omega_{0}t \end{array}$$

Equation (12) shows that when the resonator spherical shell is in the second-order vibration mode, the first and third harmonics of its mass defects also cause the hemispherical resonator gyroscope head to initiate forced vibration in the $y_{sw}$ direction.

### 2.5. Modeling of Energy Dissipation in Hemispherical Resonator Gyroscope

When the hemispherical resonator gyroscope head is subjected to forced vibration in direction $x_{sw}$, the impulse transferred to the hemispherical resonator gyroscope head by the spherical shell in half a vibration cycle is:

(13) $$\begin{array}{cc} I_{x_{sw}}\; & =\int_{0}^{\frac{\pi }{\omega_{0}}}F_{x_{sw}}dt\\ & =-\frac{A_{l}\omega_{0}\pi \rho_{0}hR_{m}^{2}}{2}\int_{0}^{\frac{\pi }{2}}\left[U\left(\alpha \right)\cos\alpha -V\left(\alpha \right)+W\left(\alpha \right)\sin\alpha \right]\sin\alpha d\alpha \cdot \\ & \left[3\varepsilon_{1}\cos\left(2\vartheta -\beta_{1}\right)+\varepsilon_{3}\cos\left(2\vartheta -3\beta_{3}\right)\right] \end{array}$$

When the resonator hemispherical resonator gyroscope head is in the oscillating state, the energy transferred from the spherical shell to the hemispherical resonator gyroscope head in one vibration cycle is:

(14) $$\begin{array}{cc} W_{I_{x_{sw}}}\; & =\frac{I_{x_{sw}}^{2}}{M_{s}}\\ & =\frac{\pi^{2}A^{2}\omega_{0}^{2}\rho_{0}^{2}h^{2}R_{m}^{4}}{4M_{s}}\left[\begin{array}{l} 9\varepsilon_{1}^{2}\cos^{2}\left(2\vartheta -\beta_{1}\right)\\ +6\varepsilon_{1}\varepsilon_{3}\cos\left(2\vartheta -\beta_{1}\right)\\ \cos\left(2\vartheta -3\beta_{3}\right) \end{array}\right.\left.+\varepsilon_{3}^{2}\cos^{2}\left(2\vartheta -3\beta_{3}\right)\right]\cdot \\ & {\left[\int_{0}^{\frac{\pi }{2}}\left[\begin{array}{l} U\left(\alpha \right)\cos\alpha \\ -V\left(\alpha \right)+W\left(\alpha \right)\sin\alpha \end{array}\right]\sin\alpha d\alpha \right]}^{2} \end{array}$$

where $M_{s}$ is the mass of the spherical shell of the resonator with the expression:

(15) $$M_{s}=\int_{R_{m}-\frac{h}{2}}^{R_{m}+\frac{h}{2}}\int_{0}^{\frac{\pi }{2}}\int_{0}^{2\pi }\rho \left(\beta \right)\cdot r^{2}\sin\alpha d\alpha d\beta dr\approx 2\pi \rho_{0}hR_{m}^{2}$$

Equation (14) expresses the energy of the spherical shell vibration is converted into the deformation energy of the hemispherical resonator gyroscope head. If the quality factor of the hemispherical resonator gyroscope head in the oscillating state is $Q_{I_{sw}}$, the energy consumed by the hemispherical resonator gyroscope head along the $x_{sw}$-direction in one vibration cycle of the resonator spherical shell is:

(16) $$\begin{array}{cc} \Delta W_{I_{x_{sw}}}\; & =\frac{2\pi W_{I_{x_{sw}}}}{Q_{I_{sw}}}\\ & =\frac{\pi^{3}A^{2}\omega_{0}^{2}\rho_{0}^{2}h^{2}R_{m}^{4}}{2M_{s}Q_{I_{sw}}}\left[9\varepsilon_{1}^{2}\cos^{2}\left(2\vartheta -\beta_{1}\right)+6\varepsilon_{1}\varepsilon_{3}\cos\left(2\vartheta -\beta_{1}\right)\cos\left(2\vartheta -3\beta_{3}\right)\right.\\ & \left.+\varepsilon_{3}^{2}\cos^{2}\left(2\vartheta -3\beta_{3}\right)\right]\cdot {\left[\int_{0}^{\frac{\pi }{2}}\left[U\left(\alpha \right)\cos\alpha -V\left(\alpha \right)+W\left(\alpha \right)\sin\alpha \right]\sin\alpha d\alpha \right]}^{2} \end{array}$$

Similarly, the energy consumed by the hemispherical resonator gyroscope head along the $y_{sw}$-direction in one vibration cycle of the resonator spherical shell is:

(17) $$\begin{array}{cc} \Delta W_{I_{y_{sw}}}\; & =\frac{2\pi W_{I_{y_{sw}}}}{Q_{I_{sw}}}=\frac{\pi^{3}A^{2}\omega_{0}^{2}\rho_{0}^{2}h^{2}R_{m}^{4}}{2M_{s}Q_{I_{sw}}}\\ & \left[9\varepsilon_{1}^{2}\sin^{2}\left(2\vartheta -\beta_{1}\right)-6\varepsilon_{1}\varepsilon_{3}\sin\left(2\vartheta -\beta_{1}\right)\sin\left(2\vartheta -3\beta_{3}\right)\right.\\ & \left.+\varepsilon_{3}^{2}\sin^{2}\left(2\vartheta -3\beta_{3}\right)\right]\cdot {\left[\int_{0}^{\frac{\pi }{2}}\left[U\left(\alpha \right)\cos\alpha -V\left(\alpha \right)+W\left(\alpha \right)\sin\alpha \right]\sin\alpha d\alpha \right]}^{2} \end{array}$$

When the resonator spherical shell containing the first harmonic and third harmonic of the mass defect is operating in the second order vibration mode, the internal dissipation of the hemispherical resonator gyroscope head to the hemispherical shell is:

(18) $$\begin{array}{cc} \frac{1}{Q_{Sup\_I_{sw}}}\; & =\frac{\Delta W_{I_{x_{sw}}}+\Delta W_{I_{y_{sw}}}}{2\pi E_{k_{\max}}}\\ & =\frac{\pi^{2}A^{2}\omega_{0}^{2}\rho_{0}^{2}h^{2}R_{m}^{4}}{4M_{s}Q_{I_{sw}}E_{k_{\max}}}\left[9\varepsilon_{1}^{2}+\varepsilon_{3}^{2}+6\varepsilon_{1}\varepsilon_{3}\cos\left(4\vartheta -\beta_{1}-3\beta_{3}\right)\right]\cdot \\ & {\left[\int_{0}^{\frac{\pi }{2}}\left[U\left(\alpha \right)\cos\alpha -V\left(\alpha \right)+W\left(\alpha \right)\sin\alpha \right]\sin\alpha d\alpha \right]}^{2} \end{array}$$

When the hemispherical resonator gyroscope head makes a forced vibration in the direction $z_{sw}$, the impulse transferred to the hemispherical resonator gyroscope head by the resonator spherical shell in half a vibration cycle is:

(19) $$\begin{array}{cc} I_{z_{sw}}\; & \approx \int_{0}^{\frac{\pi }{\omega_{0}}}F_{z_{sw}}dt\\ & =A\omega_{0}\pi \varepsilon_{2}\rho_{0}hR_{m}^{2}\cos\left(2\vartheta -2\beta_{2}\right)\int_{0}^{\frac{\pi }{2}}\left[U\left(\alpha \right)\sin\alpha -W\left(\alpha \right)\cos\alpha \right]\sin\alpha d\alpha \end{array}$$

When the hemispherical resonator gyroscope head is in a state of tensile vibration, the energy transferred to the hemispherical resonator gyroscope head by the resonator spherical shell in one vibration cycle is:

(20) $$\begin{array}{cc} W_{I_{z_{sw}}}\; & =\frac{I_{z_{sw}}^{2}}{M_{s}}\\ & =\frac{A^{2}\omega_{0}^{2}\pi^{2}\rho_{0}^{2}h^{2}R_{m}^{4}}{2M_{s}}{\left[\int_{0}^{\frac{\pi }{2}}\left[U\left(\alpha \right)\sin\alpha -W\left(\alpha \right)\cos\alpha \right]\sin\alpha d\alpha \right]}^{2}\cdot \\ & \left[\varepsilon_{2}^{2}+\varepsilon_{2}^{2}\cos\left(4\vartheta -4\beta_{2}\right)\right] \end{array}$$

Equation (20) reveals that part of the vibration energy of the spherical shell is converted into the deformation energy of the hemispherical resonator gyroscope head. If the quality factor of the hemispherical resonator gyroscope head in the stretched state is $Q_{I_{st}}$, the energy consumed by the hemispherical resonator gyroscope head along the $z_{sw}$-direction in one vibration cycle of the resonator spherical shell is:

(21) $$\begin{array}{cc} \Delta W_{I_{z_{sw}}}\; & =\frac{2\pi W_{z_{sw}}}{Q_{I_{st}}}\\ & =\frac{A^{2}\omega_{0}^{2}\pi^{3}\rho_{0}^{2}h^{2}R_{m}^{4}}{M_{s}Q_{I_{st}}}{\left[\int_{0}^{\frac{\pi }{2}}\left[U\left(\alpha \right)\sin\alpha -W\left(\alpha \right)\cos\alpha \right]\sin\alpha d\alpha \right]}^{2}\cdot \\ & \left[\varepsilon_{2}^{2}+\varepsilon_{2}^{2}\cos\left(4\vartheta -4\beta_{2}\right)\right] \end{array}$$

When the resonator spherical shell containing the second harmonic of the mass defect operates in the second order vibration mode, the internal dissipation of the hemispherical resonator gyroscope head to the hemispherical shell is:

(22) $$\begin{array}{cc} \frac{1}{Q_{Sup\_I_{st}}}\; & =\frac{\Delta W_{I_{z_{sw}}}}{2\pi E_{k_{\max}}}\\ & =\frac{A^{2}\omega_{0}^{2}\pi^{2}\rho_{0}^{2}h^{2}R_{m}^{4}}{2M_{s}Q_{I_{st}}E_{k_{\max}}}{\left[\int_{0}^{\frac{\pi }{2}}\left[U\left(\alpha \right)\sin\alpha -W\left(\alpha \right)\cos\alpha \right]\sin\alpha d\alpha \right]}^{2}\cdot \\ & \left[\varepsilon_{2}^{2}+\varepsilon_{2}^{2}\cos\left(4\vartheta -4\beta_{2}\right)\right] \end{array}$$

Equations (18) and (22) indicate that the first three harmonics of the quality defect in the resonator shell will increase the non-uniformity of the quality factor.

## 3. Results and Discussion

### 3.1. Amplitude-Phase Control System

**Figure 4:**
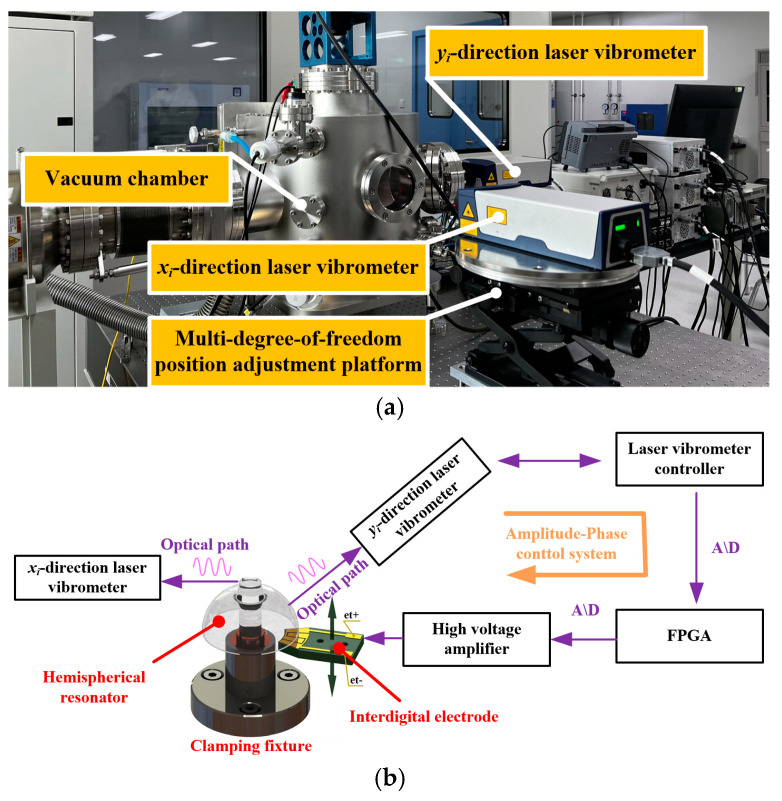
System diagram of the circumferential non-uniform quality factor of the hemispherical resonator. (**a**) A testing system for circumferential non-uniformity of resonator quality factor. (**b**) Schematic diagram of resonator testing.

As shown in Figure 4, the schematic diagram of the circumferential non-uniform quality factor testing process of the hemispherical resonator cavity is as follows:

(1) Use clamping fixtures to fix the support rod of the resonator, and adjust the relative posture between the interdigital electrode and the lip end face of the resonator shell.
(2) The measurement optical path of the Doppler laser vibrometer in the $x_{i}$ direction is focused on the center position of the outer cylindrical surface of the support rod on the resonator, and the measurement optical path of the Doppler laser vibrometer in the $y_{i}$ direction is focused on the position of the lip edge of the outer spherical surface of the resonator. The $y_{i}$ direction vibrometer is used for amplitude control, while the $x_{i}$ direction vibrometer measures the amplitude of the resonator support rod.
(3) Start the vacuum system, and the control system is by controlled controlling the mechanical pump, the speed of the molecular pump, and the gas circuit switch to safeguard the vacuum degree in the vacuum chamber down to below 5 × 10^−5^ Pa.
(4) Turn on the vacuum rotary table so that it drives the resonator to rotate around its own rotary axis to the target angular position. Then start the amplitude-phase control system of the resonator, and record the amplitude of the lock-in amplifier output after the resonator amplitude reaches a stable state.
(5) Disconnect the amplitude-phase control system of the resonator, the direction of the Doppler laser vibrometer outputs the vibration rate signal of the resonator spherical shell, and the data processing computer calculates the quality factor of the resonator according to the vibration parameter identification algorithm based on the spectral analysis method and the envelope correction analysis method.
(6) After completing the testing of all target angular positions of the resonator, the data processing computer calculates the index of non-uniformity of the resonator quality factor based on the resonator quality distribution identification algorithm.

### 3.2. Hemispherical Resonator Gyroscope Vibration Performance Test System

**Figure 5:**
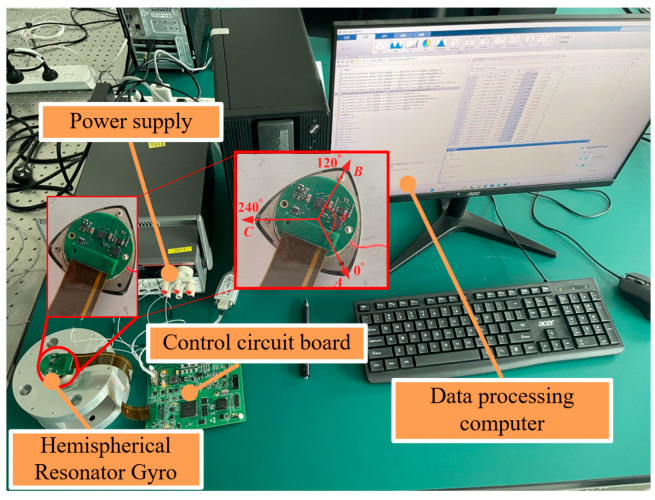
Hemispherical resonator gyroscope testing system.

As shown in Figure 5, the experimental test platform is built, which mainly includes a data processing computer, a hemispherical resonator gyroscope, a power supply and a control circuit board. In the experiment, the HRG was installed and fixed on the experimental platform by tightening bolts at positions A, B, and C. Activate the resonator control system to sequentially drive the resonator standing waves to various target angular positions, then disconnect the amplitude, orthogonality, and standing wave azimuth control circuits of the gyroscope. At the same time, collect the secondary demodulation data of the gyroscope at each standing wave azimuth and perform square root operation on it. The data processing computer calculates the mechanical time constant of each target angular position in the circumferential direction of the resonator based on the attenuation method, and calculates the quality factor non-uniformity of the resonator based on the damping distribution identification algorithm on this data.

### 3.3. Experimental Test Results

In the experimental section of this article, detailed testing and analysis were conducted on resonator #1 and resonator #2 samples before assembly. Subsequently, these two harmonic resonators were assembled into hemispherical resonator gyroscope #1 and hemispherical resonator gyroscope #2, respectively, and further testing and analysis were conducted on them.

**Figure 6:**
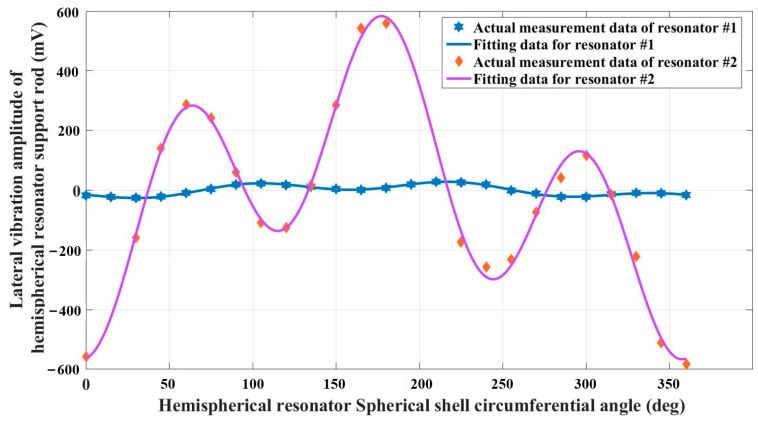
The first three test results of resonator quality defects.

As shown in Figure 6, the first three quality defect testing experiments were conducted on the samples of resonator #1 and resonator #2. The horizontal axis represents the circumferential angle of the hemispherical resonator shell, and the vertical axis represents the lateral vibration amplitude of the hemispherical resonator support rod. It can be seen that the vibration amplitude of resonator #2 changes significantly more than that of resonator #1. Therefore, the greater the significant damping non-uniformity in the first three amplitude measurements of the resonator.

**Figure 7:**
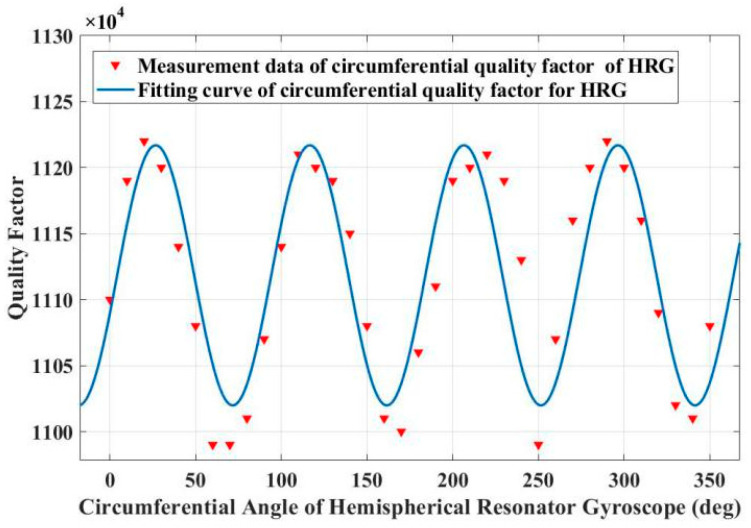
Test results of circumferential quality factor of resonator #1 before assembly.

➀ Before assembly, according to Figure 4, the measurement result of the quality factor of the resonator will not only be affected by the temperature inside the vacuum chamber, but also by the vacuum degree. Figure 7 shows the uneven quality factor test results of the resonator. From the figure, it can be seen that the quality factor exhibits a typical fourth harmonic distribution along the circumference of the resonator shell. The measured non-uniformity of the quality factor is 2.1%.

**Figure 8:**
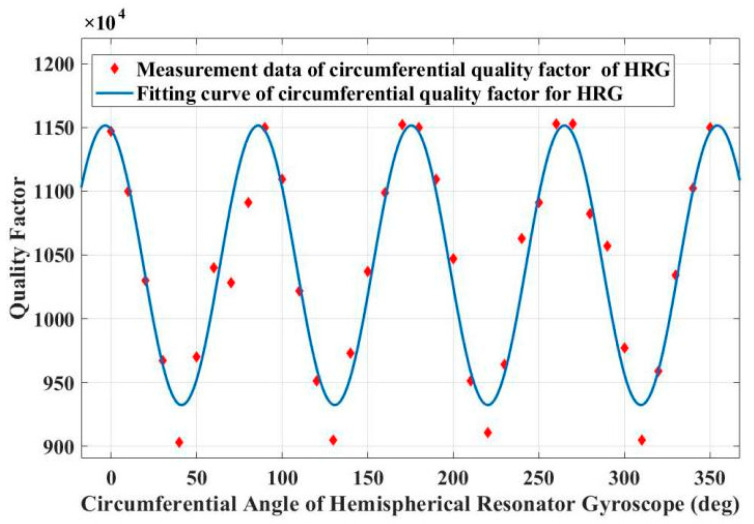
Test results of circumferential quality factor of resonator #2 before assembly.

Figure 8 shows the uneven quality factor test results of the resonator. From the figure, it can be seen that the quality factor exhibits a typical fourth harmonic distribution along the circumference of the resonator shell. The measured non-uniformity of the quality factor is 24.3%.

➁ After assembly, Group 1: hemispherical resonator gyroscope #1 uses a torque wrench to tighten the bolts in positions A, B and C with a consistent force of 0.05 N, ensuring that the circumferentially distributed force is completely symmetrical. Group 2: hemispherical resonator gyroscope #1 uses a torque wrench to tighten bolts in positions A, B, and C with a consistent force of 0.1 N. Group 3: hemispherical resonator gyroscope #1 uses a torque wrench to tighten the bolts in positions A, B, and C with a consistent force of 0.15 N.

**Figure 9:**
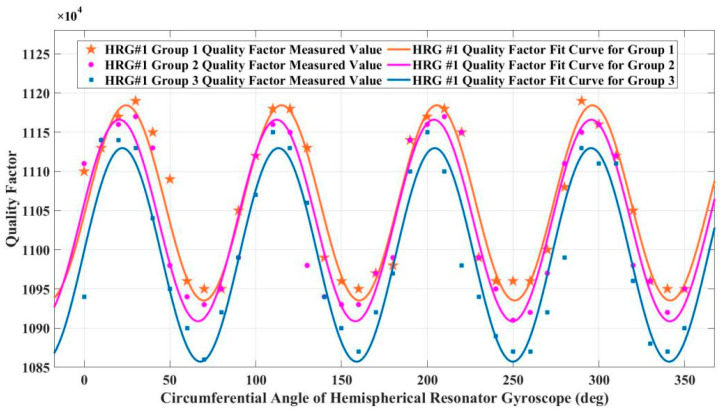
Gyroscope #1 circumferential figure of merit test results under three sets of conditions after assembly.

The measured hemispherical resonator gyroscope #1 circumferential figure of merit test results are shown in Figure 9, and the hemispherical resonator gyroscope #1 measured circumferential figure of merit inhomogeneity is 2.2% under the first set of conditions. In the second set of conditions, hemispherical resonator gyroscope #1 measured a non-uniformity of 2.4% of the circumferential figure of merit. In the third set of conditions, hemispherical resonator gyroscope #1 measured a non-uniformity of 2.6% of the circumferential figure of merit.

After assembly, Group 1: hemispherical resonator gyroscope #2 uses a torque wrench to tighten the bolts in positions A, B and C with a consistent force of 0.05 N, ensuring that the circumferentially distributed force is perfectly symmetrical. Group 2: Hemispherical resonator gyroscope #2 uses a torque wrench to tighten bolts in positions A, B, and C with a consistent force of 0.1 N. Group 3: hemispherical resonator gyroscope #2 uses a torque wrench to tighten the bolts in positions A, B, and C with a consistent force of 0.15 N.

**Figure 10:**
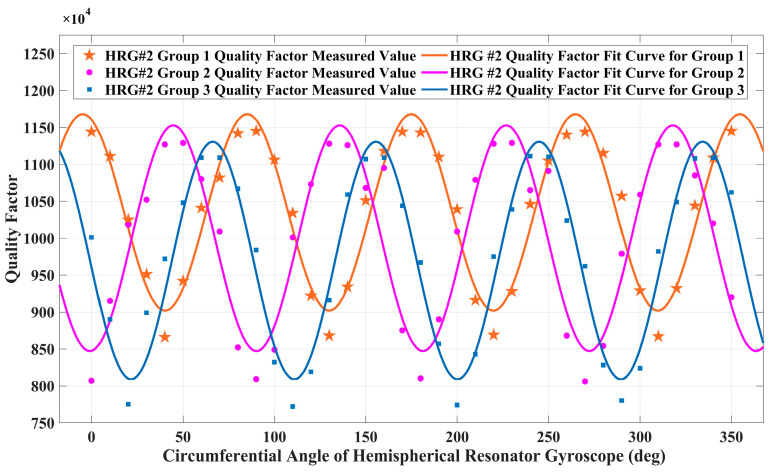
Gyroscope #2 circumferential figure of merit test results under three sets of conditions after assembly.

The measured results of the hemispherical resonator gyroscope #2 circumferential figure of merit are shown in Figure 10, and the hemispherical resonator gyroscope #2 has a non-uniformity of 27.7% in the circumferential merit factor measured under the first set of conditions. Under the second set of conditions, hemispherical resonator gyroscope #2 measured a non-uniformity of 33.4% of the circumferential figure of merit. In the third set of conditions, hemispherical resonator gyroscope #2 measured a non-uniformity of 36% of the circumferential figure of merit.

Experimental results: As shown in Figure 9 and Figure 10, the quality factor exhibits a typical fourth harmonic distribution along the gyroscope circumference. The circumferential non-uniformity of resonator #1 before assembly is 2.1%, and the circumferential non-uniformity of resonator #2 is 24.3%. As shown in Table 1, after assembling the gyroscope, by changing the force of the fixed position bolt, it can be seen that the unevenness of gyroscope #1 changes less, while the unevenness of gyroscope #2 changes more. When compensating for the gyroscope in the later stage, the change in gyroscope #1 is relatively small, so the compensation result is relatively good and more conducive to the gyroscope control.

## 4. Conclusions

This article first divides the gyroscope into two parts based on its basic structure and working principle to study its energy dissipation mechanism. Then, a mechanical model of the gyroscope head and resonator shell with quality defects was established, and the unbalanced force exerted by the resonator shell on the gyroscope head was analyzed. On this basis, the energy transfer path and dissipation mechanism were explained and analyzed. Finally, testing platforms for the non-uniformity of circumferential quality factors of hemispherical resonators and hemispherical resonator gyroscopes were built separately. Experiments have shown that the greater the significant damping non-uniformity of the first three amplitudes of resonator quality defects before assembly, the greater the non-uniformity of the circumferential quality factor. The small non-uniformity of the circumferential quality factor of the resonator has little effect on the stress of changing the installation position after later assembly into a gyroscope, thus verifying the rationality of the theory. This provides a reference for the development of hemispherical resonator gyroscopes with high *Q* values and low damping non-uniformity in the future.

## Figures and Tables

**Table 1:** The results of circumferential non-uniformity of hemispherical resonator gyroscope.

Applied Force	Circumferential Non-Uniformity of Quality Factor of Hemispherical Resonator Gyroscope #1	Circumferential Non-Uniformity of Quality Factor of Hemispherical Resonator Gyroscope #2
0.05 N	2.2%	27.7%
0.1 N	2.4%	33.4%
0.15 N	2.6%	36%

## Data Availability

Data are available from the corresponding author upon reasonable request.
